# Genetic architecture of self-limited delayed puberty and congenital hypogonadotropic hypogonadism

**DOI:** 10.3389/fendo.2022.1069741

**Published:** 2023-01-16

**Authors:** Valeria Vezzoli, Faris Hrvat, Giovanni Goggi, Silvia Federici, Biagio Cangiano, Richard Quinton, Luca Persani, Marco Bonomi

**Affiliations:** ^1^ Department of Endocrine and Metabolic Diseases and Lab of Endocrine and Metabolic Research, IRCCS Istituto Auxologico Italiano, Milan, Italy; ^2^ Department of Medical Biotechnology and Translational Medicine, University of Milan, Milan, Italy; ^3^ Department of Endocrinology, Diabetes & Metabolism, Newcastle-upon-Tyne Hospitals, Newcastle-upon-Tyne, United Kingdom; ^4^ Translational & Clinical Research Institute, University of Newcastle-upon-Tyne, Newcastle-upon-Tyne, United Kingdom

**Keywords:** delayed puberty, congenital hypogonadotropic hypogonadism, self-limited delayed puberty, genetic test, GnRH deficiency

## Abstract

Distinguishing between self limited delayed puberty (SLDP) and congenital hypogonadotropic hypogonadism (CHH) may be tricky as they share clinical and biochemical characteristics. and appear to lie within the same clinical spectrum. However, one is classically transient (SDLP) while the second is typically a lifetime condition (CHH). The natural history and long-term outcomes of these two conditions differ significantly and thus command distinctive approaches and management. Because the first presentation of SDLP and CHH is very similar (delayed puberty with low LH and FSH and low sex hormones), the scientific community is scrambling to identify diagnostic tests that can allow a correct differential diagnosis among these two conditions, without having to rely on the presence or absence of phenotypic red flags for CHH that clinicians anyway seem to find hard to process. Despite the heterogeneity of genetic defects so far reported in DP, genetic analysis through next-generation sequencing technology (NGS) had the potential to contribute to the differential diagnostic process between SLDP and CHH. In this review we will provide an up-to-date overview of the genetic architecture of these two conditions and debate the benefits and the bias of performing genetic analysis seeking to effectively differentiate between these two conditions.

## Introduction

### Definition and etiology

Delayed puberty (DP) is one of the most common clinical conditions evaluated by the pediatric endocrinologist, affecting over 2% of adolescents, and implies the lack of the first signs of pubertal development beyond the average expected age for the normal population or, when puberty has previously begun, the failure of appropriate progression. Due to the earlier onset of pubertal development in girls, puberty is generally defined to be delayed if there is absence of first pubertal signs at 13 years of age in females (breast budding or thelarche) and or 14 in males (testicular enlargement over 3-4 mL of volume), respectively. These limits of normality correspond to the mean age +2-2.5 SD of the healthy general population in Western Europe. This implies that 2.5-3% of the normal subjects will fall in the extreme tail of the normal gaussian curve, and will be classified as having DP although, as better explained below, they will likely fall in the self-limited (“benign”) form of this condition. Several underlying etiologies cause pubertal delay (**Table 1**), including chronic disease, energy-deficit and hypo- and (in girls) hyper- gonadotropic hypogonadism (1). To date, self-limited, or constitutional, DP (SLDP) is the highly common cause of pubertal delay in early adolescence, involving around 2/3 of male and 1/3 of female with DP; a para-physiological condition where individuals start puberty late, but eventually progressing to achieve full sexual maturation. By contrast, hypogonadism becomes the more common cause of DP by the late teenage years. Hypergonadotropic hypogonadism is due to intrinsic or primary gonadal failure and can easily be discerned from other forms of DP through unstimulated reproductive hormone levels, indicated by low sex steroids and high gonadotropins. It particularly concerns females (21% of DP cases), with Turner syndrome seen in 27% of girls with premature ovarian insufficiency (POI) (2, 3) but is a very uncommon cause of pubertal failure in males (vanishing testes syndrome). Conversely differential diagnosis between SLDP and hypogonadotropic hypogonadism (HH) is often challenging, as both conditions can exhibit overlapping clinical and hormonal features (2), albeit that more careful clinical ascertainment of CHH red flags could potentially identify around 50% of the CHH cases with a high degree of probability (4).

**Table 1:** List of conditions associated with CHH or SLDP.

	PARA-PHYSIOLOGICAL CONDITION	DISEASE CONDITION	
CLASSIFICATION	SLDP	HYPOGONADOTROPIC HYPOGONADISM	
		ORGANIC	FUNCTIONAL
**COMMON CAUSES**	Common familial component (associated genes: IGSF10, H6ST1, EAP1, LGR4, FTO)	- CHH (Kallmann or CHH, up to 60 genes with variable inheritance and expressivity - CHARGE - MPHD - Acquired lesions (e.g., infiltrative lesions, tumors) - Metabolic diseases (e.g., iron overload) - Autommune diseases (e.g., hypophisitis) - Other genetic syndromes (e.g., Prader-Willi) - Iatrogenic causes (e.g., surgery, radiotherapy)	- Chronic illness - Energy deficit (malnutrition, malabsorption, eating disorder, excessive exercise) - Stress - Drugs (opiates, cannabinoids, dopamine-antagonists and, in males, glucocorticoids) - Other endocrine diseases (e.g., hyper-PRL or male Cushing’s)
		*n.b.* Hypothalamic amenorrhoea comprosed both energy deficit and genetic elements (Delaney A et al, J Clin Endocrinol Metab. 2021 Mar 8;106(3):e1441-e1452.)	
**FREQUENCY**	1:50	1:5000-20,000	

### Clinical features

Adolescents with CHH and SLDP present similar clinical traits and hormonal status at presentation but respond very differently to “expectative management” in terms of physical development and long-term psychosexual outcomes (5, 6). DP in females is established when breast budding development is absent by the age of 13 years, while in males when the testicular volume fails to surpass the threshold of 4 mL volume (evaluated by Prader orchidometer) by the age of 14 years (7). Family history of CHH versus SLDP can be helpful, but both conditions may occur within the same family (8, 9). Regarding phenotypic red flags for CHH, these comprise reproductive and non-reproductive defects, with the former only observed in males. A history of cryptorchidism, particularly the bilateral form, together with a possible neonatal underdeveloped penis (2.5 SD below average size for healthy subjects) and/or hypospadias in males is strongly suggests pre- and post-natal gonadotropin deficiency characteristic of CHH (absent minipuberty). Indeed, a definitive diagnosis can be made through basal biochemical evaluation of the HPG axis during the postnatal weeks corresponding to normal minipuberty (10, 11). However, there are no external signs postnatally in CHH female CHHs to suggest a congenital GnRH secretion defect. However, non-reproductive CHH red flags occur in both sexes, comprising clefting of the palate and/or lips, olfactory defects, alteration of digital bones, hearing loss, colorblindness, nystagmus and bimanual synkinesis, renal and/or dental agenesis or dysgenesis (10, 11). Anosmia or hyposmia are reported in around 50% of patients with CHH, thus olfactory defects (evaluated with T2-weighted coronal MRI of the olfactory bulbs and sulci and/or a quantitative olfactory test) should be evaluated and, whenever present, considered as a reliable clue towards CHH diagnosis (12). Slow growth rate and low weight tend to suggest functional HH or SLDP (7), especially when syndromic manifestations or red flags are absent. In contrast, children with CHH exhibit regular linear growth, although delayed bone maturation, osteopenia, and osteoporosis may be observed when CHH is diagnosed later in life (13, 14).

### Hormone and stimulation test for differential diagnosis

Unlike the postnatal period, gonadotropin and sex hormone measurements cannot discriminate between SLDP and CHH in early adolescence, because gonadotropin concentrations are frequently borderline also in healthy subjects of this age (1). Frequent overnight blood sampling and LH pulse-analysis has been considered for the differential diagnosis, although similar pulsatile “fluctuation” was observed in patients with SLDP, CHH and in normal prepubertal children (15). Moreover, this technique can’t realistically be into routine clinical practice. In addition, the diagnostic efficacy of a single basal gonadotropin concentration it is inadequate (16, 17). A similarly low diagnostic power is offered by dynamic gonadotropin testing with GnRH or GnRH analogs (18). In fact, the lack of a gonadotropin reaction to GnRH stimulation can only confirm the absence of puberty onset, but it is not sufficient to provide a true differential diagnosis between SLDP and CHH (19). Measurement of Inhibin B, Anti-Mullerin Hormone, AMH, INSL3, and testosterone after hCG stimulation have been proposed to guide differential diagnosis (20). Although the data are promising (18, 21), they are not sufficient to support the clinical value of these newer endocrine markers. The use of new ultrasensitive methods (LC/MS) for determining hormone levels might, however, unravel new diagnostic perspectives. The testosterone response to long-term hCG stimulation and peak serum FSH response to GnRH were found to be significantly different in CHH patients (22), but there are potential long-term drawbacks to long-term hCG therapy in males who are FSH-naïve, in terms of promoting differentiation of a limited pool of Sertoli and germ cells before they have a chance to proliferate under FSH stimulation. Moreover, these methods are too inconvenient and expensive to be useful first-line approaches. Nevertheless, hormonal responsiveness to kisspeptin in boys with delayed puberty appears to be a promising new hormonal marker, although currently further studies are needed (23).

### Low dose sex steroid “priming” test

Recent studies have examined the diagnostic value of testosterone priming to differentiate between SLDP from CHH. Short-term testosterone therapy (oral, injections or transdermal) in boys with SLDP would prompt HPG activation, with the result of enlargement of the testis and rise in the production of endogenous testosterone (24). This proposed “diagnostic test” would speed up diagnosis and consequent treatment of SLDP patients, with benefits on growth velocity and virilization (25). Subjects not responding to testosterone priming (i.e., CHH patients) (24) could be than efficiently analyzed with the more expensive tests described above or offered more sustained testosterone treatment. Estradiol priming has also been proposed for HPG activation in SLDP females (2). Although, as discussed in previous paragraphs, clinicians could benefit of multiple clinical and biochemical tests to produce the diagnosis, none of these could accurately discriminate between those patients who will naturally pass and progress normally during puberty (i.e. SLDP) and those who will likely require lifelong medical therapy (i.e. HH) (2, 3). The clinical distinction between SLDP and CHH in early adolescence is of crucial importance as if CHH is diagnosed, prompt drug treatment is mandatory for puberty induction (26–28), with combined gonadotropin therapy having the potential to optimize fertility potential and quality of life in males (29).

## Genetics of CHH

**Figure 1:**
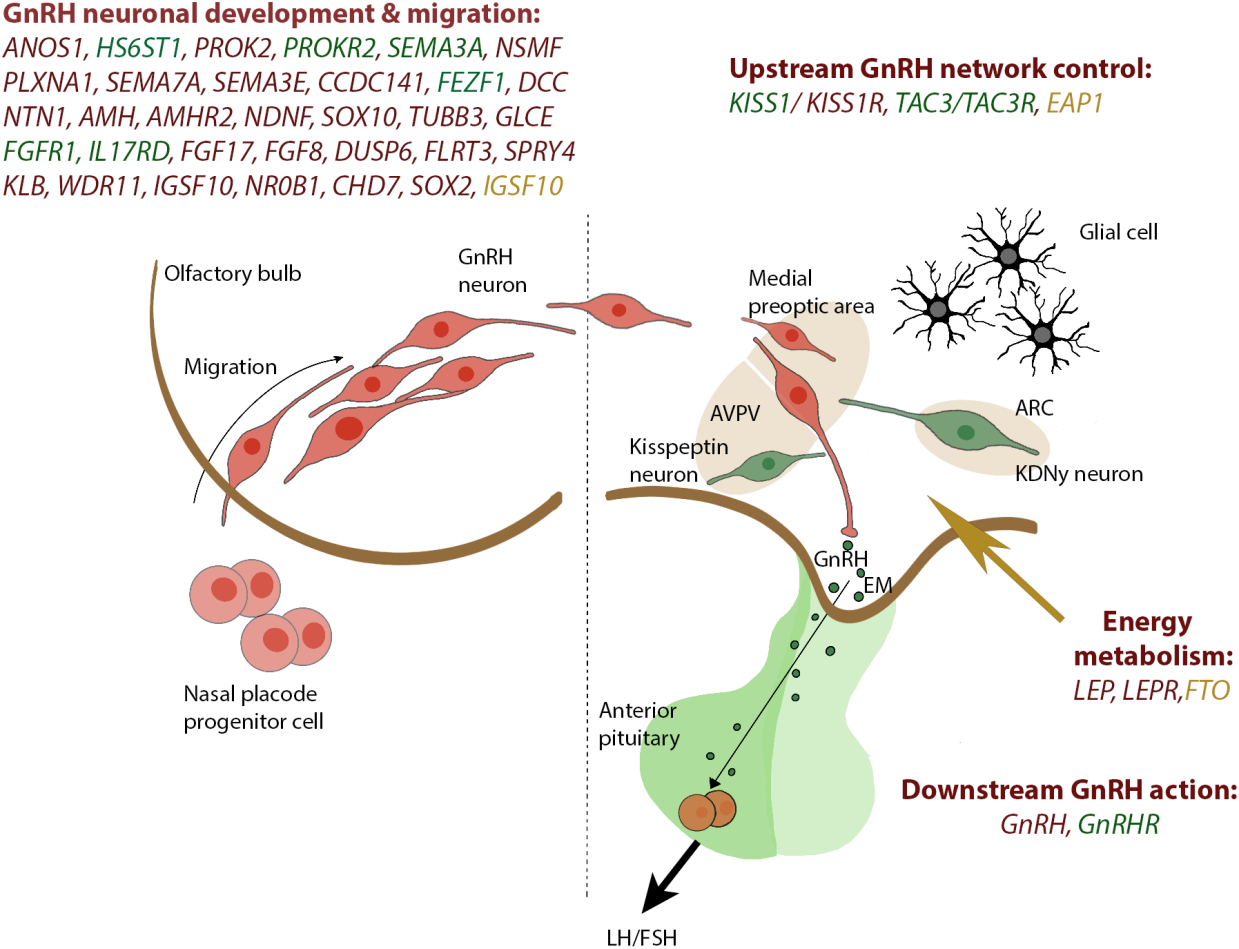
Genes reported in CHH and SLDP are related to GnRH development and GnRH function. The drawing on the left panel shows the migration of the GnRH neurons from the olfactory placode and into the hypothalamus which occurs within the first 10 to 12 weeks of rodent life. In the right panel, a concise view of our current understanding of the KISS1 neuronal networks governing GnRH secretion is depicted. Genes associated with CHH are depicted in red, shared genes between CHH and SLDP are depicted in green whereas genes related to SLDP are marked in orange. GnRH, gonadotropin-releasing hormone; KNDy, Kiss1/NKB/Dyn neuron; ARC, arcuate nucleus; AVPV, anteroventral periventricular nucleus; EM, median eminence.

CHH is defined by the diagnosis of gonadotropic deficiency throughout the infant mini- puberty or when puberty is absent or arrested in adolescence (30), although the median age at diagnosis and effective treatment of CHH remains unacceptably high around 19 years (5) and many patients present much later in adult life with sexual dysfunction, infertility, anaemia, myopathy or osteoporosis (31). CHH accounts for 24 to 85% of stable hypogonadotropic hypogonadism and includes normosmic subjects (nCHH) and subjects with olfactory defects identifying the Kallmann Syndrome (KS). KS results from mutations in genes acting in the development of olfactory neurons; nCHH can also be underpinned by the same “neurodevelopmental” genes but is more commonly associated with mutations of genes that regulate GnRH secretion. Currently, the utility of this dichotomous division in targeting genetic testing is reduced by the clinical and genetic overlap between the two conditions (32). In CHH, a genetic basis can be identified in around 50% of patients (14, 33, 34), although as more genes are identified it has become apparent in many patients that the genetic variant originally believed to fully explain their condition did not in fact represent the whole story. So far, mutations in more than 60 genes have been classified as genetic cause of CHH, whether nCHH, KS, or both (**Table 2**), with few rare loci also involved in complex syndromes such as CHARGE (35–38).These genes include *ANOS1*, FGF receptor 1 (*FGFR1*), FGF8, prokineticin 2 (*PROK2*), prokineticin 2 receptor (*PROKR2*), CHD7, NMDA receptor synaptonuclear signaling and neuronal migration factor (*NSMF*), *GnRH1*, GnRH receptor (*GnRHR*), *KISS1*, *KISS1R*, tacykinin 3 (*TAC3*), *TACR3*, semaphorin 3A (*SEMA3A*), SRY-box 10 (*SOX10*), IL-17 receptor D (*IL17RD*), FEZ family zinc finger 1 (*FEZF1*), WD repeat domain 11 (WDR11), heparin sulfate 6-O-sulfotransferase 1 (*HS6ST1*), and *FGF17*. These are key genes for regulating GnRH neuronal migration and differentiation, GnRH secretion, or its upstream or downstream pathways (**Figure 1**). GnRH neuroendocrine cells originate in the olfactory placode outside the central nervous system and subsequently migrate into the brain during embryonic development (39). This route offers a developmental connection between the sense of smell and the central control of reproduction, which are both affected in Kallmann syndrome. Evidence obtained in the past years (40) suggest that GnRH neurons originating from the neural crest and ectodermal progenitors migrate in tight association with growing axons of olfactory and terminal nerves. Reached the hypothalamus, GnRH neurons finally detach from their TN guiding fibers, disperse further into the brain parenchyma, and stop the migration. At birth, GnRH neurons project to the hypothalamic median eminence and release GnRH into the hypophyseal portal vasculature (41). GnRH acts *via* the GnRH receptor, which is expressed on gonadotropic cells in the anterior pituitary gland. This action elicits the secretion of the gonadotropins, luteinizing hormone and follicle-stimulating hormone which control gonadal maturation and adult reproductive physiology *via* the hypothalamic–pituitary–gonadal (HPG) axis. As with many other diseases of genetic origin, the application of next generation sequencing technology (NGS) to the diagnosis of CHH has boosted the discovery of new candidates involved in its etiology. All known forms of inheritance have been described: autosomal dominant, recessive and with variable penetrance; X-linked recessive; oligogenicity, and transmission linked to an imprinting locus. Moreover, as already reported for Fgf8 signaling system (42, 43), also interferences in the pre-hypothalamic epigenome (throught DNMTs/TETs proteins) could have major consequences on GnRH system neurodevelopment, resulting in CHH disorder. Due to the variable expressivity and incomplete penetrance of the genetic defects, together with the actual or potential impact of oligogenicity and epigenome modifications, there is a broad spectrum of phenotypes, whether with non-reproductive defects or pure neuroendocrine phenotype, and ranging from complete CHH, with LH/GnRH apulsatility and absent pubertal development (around 2/3 of cases), to partial hypogonadism with residual LH/GnRH pulsatility (low amplitude, low frequency, or nocturnal-only pattern) resulting in arrested early puberty (around 1/3 of cases), and even reversible CHH in 5 to 20% of cases patients (44). Crucially, the genetic architecture of CHH as is presently understood does not explain the 3-5-fold excess of affected males.

**Table 2:** List of genes implicated in CHH.

Gene symbol	OMIM	Inherit-ance	Olfactory defect	Main phisiological mechanism	Functionally validated variants
LEP	164160	AR	nCHH	Mimics energy-deficit HH in the face or early-onset morbid obesity	X
LEPR	601007	AR	nCHH		X
GnRH1	152760	AR, olig	nCHH	GnRH function	X
GnRHR	138850	AR, olig	nCHH		X
KISS1	603286	AR	nCHH	GnRH neuron activatio	X
KISS1R	604161	AR	nCHH		X
TAC3	162330	AR	nCHH		X
TACR3	162332	AR, olig	nCHH		X
ANOS1	300836	XLR	KS	GnRH migration	X
HS6ST1	604846	Olig	KS or CHH		X
PROK2	607002	AR,AD,olig	KS or nCHH		X
PROKR2	607123	AR,AD,olig	KS or nCHH		X
SEMA3A	603961	AD,olig	KS or nCHH		X
PLXNA1	601055	AR, olig	KS or nCHH		
SEMA7A	607961	olig	KS or nCHH		
SEMA3E	608166	olig	KS or nCHH		X
NSMF	608137	AR,olig	KS or nCHH		X
CCDC141	616031	AR, olig	nCHH		
FEZF1	613301	AR	KS		X
DCC	120470	AD, olig	KS or nCHH		X
ntn1	601614	AD, olig	KS or nCHH		X
AMH	600957	AD	KS or nCHH		X
AMHR2	600956	AD	KS or nCHH		X
NDNF	616506	AD	KS		X
SOX10	602229	AD	KS		X
TUBB3	602661	AD	KS		X
GLCE	612134	–	KS or nCHH		
FGFR1	136350	AD	KS or nCHH	GnRH neuron fate specification	X
IL17RD	606807	Olig	KS or nCHH		X
FGF17	603725	Olig	KS or nCHH		X
FGF8	600483	Olig	KS or nCHH		X
DUSp6	602748	Olig	KS or nCHH		
FLRT3	604808	Olig	KS or nCHH		
sPRY4	607984	Olig	KS or nCHH		
KLB	611135	AD	KS or nCHH		X
WDR11	606417	AD, olig	KS or nCHH		X
IGSF10	617351	AD	nCHH		X
NR0B1	300473	XLR	nCHH		X
CHD7	608892	AD, AR, olig	KS or nCHH		X
SOX2	184429	AR	nCHH		X

CHH, congenital hypogonadotropic hypogonadism; KS, Kallmann syndrome; AD, autosomal dominant; AR, autosomal recessive; olig: oligogenic. X, yes.

### Autosomal recessive forms

Isolated CHH is transmitted as an autosomal recessive trait due to mutations in three genes: *GnRHR*, for which over 60 families have been reported (45–49), *KISS1R*, with 27 identified families (45, 50) and *TACR3*, found in 20 families (45, 51–54). In these patients, the phenotype is a common nCHH, without any non-reproductive traits of disease (36). It is worth to report that for *GnRHR* mutations, responsible for about 40-50% of hereditary cases of nCHH (48), there is a broad phenotypic variability even in the same pedigree with the same mutation (55). On the other hand, biallelic mutations in *GNRH1*, *KISS1*, and *TAC3*, ligands of the above-mentioned receptors, are a rare cause of nCHH (36). Mutations in *GNRHR* represent a paradigm of impaired functionality of GnRH, in fact, *GnRHR* encodes a receptor coupled to G proteins which determines the release of gonadotropins in the pituitary (41). *KISS1R*, *TACR3* and *TAC3* genes, encoding for a G-protein-coupled receptor for Kisspeptin, for neurokinin B and its ligand, are all instead members of a complex network where the KDNY neurons exert regulatory action on GnRH function (37).

### X-linked forms

*ANOS1* (OMIM 300836), formerly *KAL1*, is characterized by an X-linked recessive inheritance pattern; intragenic microdeletions or pathological variations of this gene have been described in 10-20% of KS (33). For canonical clinical manifestations, such as CHH and anosmia, penetrance is complete (56–61). On the contrary, other clinical manifestations such as synkinesis (56) and renal agenesis show different expressions in individuals also carrying the same variant (57–68). About 144 families have been reported so far. It is worth mentioning a female phenotype observed in 10 patients, of which one case was linked to a biallelic variant of *ANOS1* (69) and the other nine cases were traced to a mutation in another gene, indicationg a possible oligogenic inheritance. *ANOS1* encodes Anosmin1, an extracellular protein that mediates cell adhesion and play a crucial role in the migration of GnRH neurons (41).

### Autosomal dominant forms

Autosomal dominant (AD) transmission of CHH is seen more often in KS than in nCHH, with the most common genes (causing both nCHH and KS) being *FGFR1* and *CHD7*. *FGFR-1* encodes a tyrosine kinase receptor that regulates central developmental processes such as neuronal migration, fate, determination, and cell proliferation (41). *FGFR1* plays a fundamental action in the proliferation and migration of GnRH neurons to the hypothalamus as well as directly promoting olfactory bulb development. Up to now, more than 140 mutations in this gene have been described, which generally lead to a loss of function with various mechanisms (splicing, nonsense, missense, frameshift and deletions) (56, 70, 71). *FGFR1* mutations related to KS are exemplified by incomplete penetrance (30, 72, 73) and variable clinical expression even in the same family, with patients presenting anosmia, complete phenotype, or isolated pubertal delay (72–75). Additionally, it was reported that mutations in this gene cause also nCHH (73, 76–78). Other clinical traits of *FGFR1* mutations, such as cleft lip and cleft palate, skeletal anomalies, and dental agenesis, are reported with variable frequency (30, 56, 66, 72, 79). With the discovery of other gene mutations in patients previously considered to have pure AD CHH due to FGFR1 mutations, it has become increasingly apparent that variable penetrance or oligogenicity are more common than AD inheritance with *FGFR1* mutations. *CHD7*, located in 8q12.1, encodes chromodomain helicase DNA-binding protein 7, which is expressed in different fetal tissues including the developing brain. It is one of two CHH genetic loci shared with CHARGE syndrome, a rare disorder with autosomal dominant inheritances, characterized by congenital heart disease, coloboma, choanal atresia, genital and ear anomalies, and growth and developmental retardation. Its pattern of expression reflects a potential *CHD7* contribution in the development of the olfactory bulb and GnRH neurons (41). Whereas CHARGE patients tend to harbor large *de novo* gene deletions, patients with KS and nCHH (80–82) tend to harbor missense mutations with partial loss of function that may either be *de novo* or inherited (83). Even in the case of *CHD7* mutations there is broad phenotypic variability, going from KS through nCHH to isolated anosmia (82, 83). Other clinical traits associated with *CHD7* mutations are deafness, anomalies of the outer ear and lip/cleft palate (83, 84). Indeed, it can sometimes be hard to distinguish between “mild CHARGE” and KS with multiple developmental defects. *SOX10* is a transcription factor crucial for the early development of neural crest cells, which are multipotent precursor arising from the neural tube that differentiate into different cell types. *SOX10* influence also hearing through its expression in the melanocytic intermediate cells of the cochlear stria vascularis during early development of the inner ear (85). AD *SOX10* mutations have been described in nearly 40% of KS patients with deafness (86). *PROKR2*, a gene encoding a G protein-coupled receptor and its ligand *PROK2* deserve to be reported in this section as a paradigm of mixed inheritance. The binding of *Prok2* to its receptor activates a signaling cascade with effects on the migration of GnRH neurons (41). Mutation in *PROKR2* were reported both in KS and nCHH. In 20% of cases AR inheritance pattern is reported for this gene, whereas the remaining cases are due to AD or oligogenic mechanisms (79, 87–94). *PROK2* variants, usually less frequent, can present with AD or AR inheritance patterns. As with *FGFR1*, some cases of *PROK2* and *PROKR2* mutations that were originally believed to represent AD inheritance have since turned out to be more probably oligogenic. *FGF8* encodes for a ligand of *FGF1* receptor (36). Heterozygous mutations of *FGF8* were identified both in KS and nCHH, also with oligogenic inheritance. The clinical signs include neurosensorial deafness camptodactyly and cleft lip/palate (3, 70, 95).

### Oligogenic inheritance

Recently in several cases of nCHH and KS a mutation in two or more genes, with oligogenic inheritance, has been reported. In 2006 a case of KS harboring mutations of both *PROKR2* and *KAL1* was reported (79). However, the authors did not fully appreciate the significance of their finding and, consequently, it was not until the following year that a convincing report of two different genetic variants in *FGFR1* and *NSMF* inherited within kindred causing KS only in the single individual carrying both (96). Subsequently, in 2010, Sykiotis (97) described oligogenic inheritance in 2.5% of subjects in a large series of CHH patients using a panel of just 8 genes. Successively an oligogenic mechanism in 7% (78) to 15% (32) of the subjects with CHH was documented by different groups. So far, an oligogenic mechanism of inheritance was documented at least for 16 genes (30). Reasonably, the application of NGS will increase the possibility of finding “oligogenicity” in CHH. However, the huge amount of data generated by NGS is now challenging the clear distinction between “oligogenicity” and the presence of benign variants that do not interfere with the phenotype that is determined by the variant that is principally responsible (36).

### CNV contribution to the genetic architecture of CHH

Even if more than 60 candidates have been linked with the pathogenesis of CHH, approximately 50% of cases remain genetically undetermined (37). Part of this missing genetic heritability probably resides in new candidates that require larger cohorts for their discovery or in mutations not simply detectable by whole exome sequencing experiments (WES), such as copy number variants (CNVs) and variants in the non-coding portion of the genome. CNVs are structural variants that result in either gain (duplications) or loss (deletions) of genetic material (more than 50 bp of genomic DNA). Thus far, previous investigation of CNVs with chromosomal microarrays or karyotypes in CHH has led to essential genetic findings (98–100), showing an overall prevalence of ~1% in a subset of CHH-associated genes (101, 102). However, such a low prevalence of CNVs in previous CHH studies has been imputed to the low-resolution tools deployed to call CNVs (101). To date, complex analytic pipelines now available can detect CNVs of a smaller size compared to historic microarrays, allowing precise characterization of these structural variants. In 2022, Balasubramanian and colleagues employed new validated, high-resolution CNV capture technology to examine a large cohort of CHH patients, detecting a total CNV prevalence of 2% across 13/62 known CHH candidates (103). Although supporting the idea that CNVs in known CHH genes should be investigated in patients with CHH, this study indicates that the greater proportion of the missing heritability in CHH may relate to mutations of new coding/non-coding genes. It is hoped that, given the massive application of genome sequencing, these still elusive variants involving non-coding regions will illuminate the full genetic architecture of CHH.

## Genetics of SLDP

Pondering the distribution of puberty timing in the normal population, SLDP can be assimilated to the extreme upper limit of normality. A clear diagnosis of this condition is often difficult to achieve, even if SLDP is frequently reported in multiple generations of the same family. Most commonly, the trait is inherited in an autosomal dominant pattern, often with complete penetrance, but autosomal recessive, X-linked and bilinear pedigrees have also been reported (104, 105). Epidemiological studies of twins in both sexes have shown that the time of puberty is a highly heritable trait and that genetics play a key role in determining when healthy individuals enter puberty (106, 107). It is clear from genome wide association studies (GWAS) that many different genetic signals are crucial in the discrepancy of pubertal timing observed in the normal population (108). In contrast, one or a small number of genetic variations in each family are generally described in delayed familial puberty, with a corresponding pattern of autosomal dominant inheritance. The recent application of NGS technology to self-limited DP have unraveled fascinating new mechanisms in the genetic control of puberty (**Figure 1**).

### HS6ST1

Targeted and whole exome sequencing methods identified a mutation in *HS6ST1* in a broad pedigree among a large cohort of patients with isolated familial delayed puberty, without associated CHH in their relatives (84). All the family members who carried the mutation exhibited a canonical SLDP phenotype, rather than CHH; the proband entering puberty spontaneously at 14.3 years. In parallel, a mouse heterozygous knockout model found the loss of an Hs6st1 allele to cause pubertal delay in females without impairment of adult reproductive capacity. Hs6st1 +/− mice showed no impairment of fertility, development of GnRH neurons, testes, or spermatogenesis. However, a substantial delay in the timing of vaginal opening (used to determine the onset of puberty in female rodents) was observed in females. Remarkably, the Hs6st1 +/- mice had normal olfactory bulbs without any reduction in the global number of GnRH neurons in the hypothalamus or projecting to the median eminence. Consequently, the pubertal delay observed in mice could be due to variation in GnRH neuron activity or other crucial downstream pathways, controlling the expression of *Hs6st1* in both the arcuate and paraventricular nucleus (109, 110). In recent work involving 338 patients with GnRH deficiency, including 105 subjects with a positive family history, a variant in HS6ST1 gene was identified in almost 2% of patients with CHH (111). In this study, the inheritance model was complex, bypassing simple Mendelian transmission, and with substantial clinical heterogeneity suggesting a role for epigenetic mechanisms or mutations in other candidates to fully explain the observed phenotypes. In order to properly assign a role to HS6ST1 in SLDP, it is crucial to take in account the biological processes in which it takes part. Howard et al., 2018, found that reduced Hs6st1 expression and a consequent reduction of sulfotransferase activity in kisspeptin and other neurons can impact on their ability to regulate GnRH function and secretion (112). In addition, *Hs6st1* activity is a prerequisite for the correct function of *Anos1* and *Fgfr1* (111). In summary, a functionally minor heterozygous mutation might cause SLDP, whereas a more severe mutation or the simultaneous effect of a second gene (i.e., oligogenicity) could lead to a more critical phenotype such as CHH or KS.

### IGFS10

Targeted and whole exome sequencing methods identified deleterious mutations also in *IGSF10* gene. Two N-terminal variants in *IGSF10* were reported in 20 subjects with SLDP from six families, with an AD inheritance (8). Moreover, two C-terminal variants in the same gene were identified in 4 families of the same study. In one family, there was incomplete penetrance, whereas a *de novo* mutation was proposed for another family. All patients had a standard growth rate before puberty and a classic DP with a delayed pubertal spurt and normal (self-reported) sense of smell. *IGFS10* gene had hitherto never been associated with any human pathology. During embryonic development mutations in *IGFS10* impact the migration of GnRH neurons from the vomeronasal organ to the forebrain. Patients with mutations in this gene are characterized by isolated pubertal delay without retardation in growth (a pattern that is also observed in CHH). Abolition of GnRH neuronal migration due to anomalous IGSF10 signaling might determine reduced or deferred migration of GnRH neurons to the hypothalamus. This result into a functional defect in the GnRH network and a higher threshold for pubertal onset. In addition, *IGSF10* loss-of-function mutations were found in subjects with hypothalamic amenorrhea-like phenotype, suggesting a common genetic origin of functional central hypogonadism with both CHH (113) and DP. Intriguingly, mutations in *IGSF10* were recently reported in a pedigree with a Kallmann- like phenotype and in patients with both disorders of neuronal development and premature ovarian insufficiency (114). Studies on the role of the *HS6ST1* and *IGSF10* genes in PD hypothesize that developmental defects in the GnRH system during fetal life may regulate the timing of onset of puberty in adolescence, without determining other associated phenotypic characteristics. Whether these patients will have any shortcomings in their long-term reproductive capacity or sex life span remains to be evaluated.

### FTO

*FTO* is considered the most impactful locus on BMI and the obesity risk (115). Recently, using NGS techniques, rare heterozygous variants in *FTO* gene associated with BMI and growth retardation in early childhood have been described in SLDP families (116). Moreover, mice lacking *FTO* had a significantly delayed onset of puberty (timing of vaginal opening) (116).

### EAP1

*EAP1* (*Enhanced at puberty 1)* encodes a nuclear transcription factor that trans-activates GnRH promoter, facilitating GnRH secretion, and parallelly inhibits the preproenkephalin promoter, which in turn represses GnRH secretion. Howard et al. first described human EAP1 mutations that appear to cause SLDP in 2 families (117). Affected subjects from these two families had canonical clinical and biochemical signs of SLDP, with delayed onset of Tanner stage 2 and delayed peak height velocity. Both subjects showed spontaneous pubertal development at age 18 without testosterone priming, thus excluding CHH. Two highly conserved variants, one rare missense variant in *EAP1* and one in-frame deletion, were identified in subjects with familial delayed puberty. Compared to wild-type EAP1, mutants showed a biased ability to transactivate the GnRH promoter, imputable to the diminished protein levels caused by the in-frame deletion and the altered subcellular localization triggered by the missense mutation. The same work showed that in monkey hypothalamus Eap1 binding to the GnRH1 promoter rise at the onset of puberty. These recent findings suggest that genes that determine SLDP may play a role in the redundant mechanisms that regulate the onset of puberty (e.g., number of cells migrating from the olfactory placode to the hypothalamus and pre-optic areas or modulation of GnRH function and secretion), despite genes linked to CHH that directly control the migration or function of GnRH neurons. However, further studies are needed to uncover the strongness of this intuition.

### LGR4

*LGR4* encodes a receptor for R-spondins which, once activated, potentiates the canonical Wnt signaling pathway. Through GWAS analysis *LGR4* had already been designed as a regulator of pubertal timing both in males (based on recalled age at voice breaking) and in females (based on the age of menarche (118, 119). However, mutations in *LGR4* were not previously associated with actual human disease. A recent study from Dunkel’s group utilizing whole-exome sequencing of 160 individuals of 67 families in a well-characterized DP cohort identified 3 rare missense variants in *LGR4* (120). All segregated with the DP trait with an AD pattern of transmission. Specific expression of *Lgr4* at the site of GnRH neuron development has been reported. LGR4 mutants showed biased Wnt/β-catenin signaling, leading to consequences on protein expression, trafficking, and degradation. Lgr4-deficient mice showed a significantly delayed onset of puberty and lowered number of GnRH neurons compared to WT mice. In addition, we were demonstrating that *lgr4* knockdown in zebrafish embryos impact development and migration of GnRH neurons. In addition, genetic lineage tracing displayed robust Lgr4-mediated Wnt/β-catenin signaling pathway stimulation during GnRH neuron development.

## Shared genes between CHH and SLDP

It is well established the timing of puberty in normal populations has a strong genetic component (107, 119, 121, 122) even though general health, nutritional status and endocrine chemical disruptor can influence the expression of key regulators. To date, the knowledge about the genetic mechanisms that control HPG axis comes largely from reports on patients with GnRH deficiency, leading to the identification of rare variants underlying CHH. Zhu and collaborators recently addressed the hypothesis of a shared genetic basis between CHH and SLDP, performing WES analysis in 15 families with a CHH proband carrying a putative pathogenic variant in CHH genes and family members both with delayed and with normal puberty (9). A genetic origin was identified in half of relatives with delayed puberty and in in a small percentage (12%) of relatives with normal puberty. Moreover, they analyzed nearly fifty DP subjects without family history of CHH matched with controls from ExAC and identified mutations in CHH genes in 14.3% of DP subjects and in 5.6% of controls. The heterozygous allelic variants were in *TAC3, TACR3, GnRHR, IL17RD* and *SEMA3A*. Of note, control subjects also carried potentially pathogenic variants. Moerover, Cassatella et al., 2018 determined that the genetic architecture of SLDP is closer to that of normal controls than CHH probands. Exome sequencing showed potentially pathogenic variant in CHH genes (twenty-five genes with IGSF10) in 51% of CHH patients, in 7% of SLDP probands and in 18% of healthy subjects. Oligogenic inheritance was found in 15% of CHH patients and in only 1.4% of SLDP subjects and 2% of controls. To note, potentially pathogenic variants in SLDP patients were found in *AXL, FGFR1, HST6ST1, PROKR2, FEZF1* and *TAC3* genes.

## Genetic evaluation supports differential diagnosis in patients with SLDP and CHH

As DP is a common condition in unaffected individuals, identifying a genetic cause in SLDP presents several pitfalls. Thus, a genetic variant likely to lead to this condition could have a quite high prevalence in the normal population. Furthermore, both the manifestation of pubertal delay in 10% of relatives of patients with CHH (9) and the likelihood of a spontaneous reversal in 10% of patients with CHH (123) remains consistent with a shared molecular basis of CHH and SLDP. Nevertheless, it remains true that genetic variants reported are distinct between the two diseases (32). This supports the hypothesis that NGS of a large panel of candidate genes might one day assist physicians to distinguish those adolescents with severe CHH from those with SLDP, enabling timely and correct treatment to CHH patients. Howard and collaborators studied the burden of genetic variants in an ethnically mixed cohort of adolescent patients with DP, with the purpose of validate the genetic analysis of known causative genes to confirm the diagnosis of CHH or SLDP. The results of this work show a fair correlation between patient genotype and clinical diagnosis, with a specificity of 100% and PPV of genetic tests for the diagnosis of patients with CHH. The authors also reported that subjects carrying homozygous or loss-of-function mutatons in CHH genes would probably have a final diagnosis of CHH. In contrast, patients presenting mutations reported only in SLDP with heterozygous inheritance were thought to be likely to have a final diagnosis of SLDP. These results confirm that WES testing can make a clear diagnosis of CHH for 17.4% of patients presenting with pubertal delay. This finding supports the implementation of genetic analysis in the clinical practice, in combination with clinical and biochemical observations, to validate the diagnosis of CHH in adolescents presenting with DP. It is worth mentioning that the same work identified in three patients with a clinical diagnosis of SLDP also pathogenic variants in other genes previously linked to CHH, namely *DMXL2, OTUD4* and *SEMA3*.

## Conclusions

Pubertal delay can be the presentation of a wide spectrum of clinical phenotypes ranging from CHH, which is a pathological condition and needs appropriate medical therapy, to SLDP, a possible para-physiological and benign condition mostly compatible with normal reproductive capacity post- puberty, to non-gonadal illness or energy deficit. Distinguishing between SLDP and CHH remains challenging and, thus far there is no clear and univocal evidence to assist clinicians in their differential diagnosis and management of SLDP and CHH. The availability of an accurate genetic test in clinical practice to discriminate between the two conditions has the potential for significant cost savings (preventing unnecessary investigations) and improvement of health and fertility outcomes for CHH patients.

Recently, the application of NGS technologies has shed light on the complex genetic mechanisms at the basis of CHH or SLDP, identifying new candidate genes and suggesting a different genetic backbone for these two conditions. This difference constitutes the main assumption on which to model a genetic tool that allows an early differential diagnosis between the two conditions. However, it should consider that so far only a few causal genes have been described in SLDP, leading to a lower pick-up rate for SLDP pathogenic variants in a putative diagnostic panel when compared with CHH mutations. Furthermore, variants contextualization in patients with oligogenic inheritance is difficult due to our lack of knowledge of variant–variant crosstalk. For these reasons, we suggest that so far only an integrated approach can increase the sensitivity and specificity in CHH diagnosis. Thus, we invite the readers to combine this type of genetic analysis with biochemical profiling (e.g., basal LH, FSH, inhibin B, AMH) and an accurate physical and anamnestic data collection to maximize the diagnostic accuracy. Nevertheless, in the future, as the knowledge of the genetic architecture of delayed puberty will be dramatically improved, genetic testing might offer a quick and precocious analytical tool also in clinical routine.

## Author contributions

VV, FH, and MB wrote the draft. MB, GG, SF, BC, RQ and LP revised the manuscript. VV and MB finalized the manuscript. All authors contributed to the article and approved the submitted version.
